# Diagnostic value of high sensitivity cardiac troponin T (hs-cTnT) in dialysis patients with myocardial infarction

**DOI:** 10.3389/fcvm.2023.1278073

**Published:** 2023-12-21

**Authors:** Kun Zhao, Bozhi Shen, Hongcheng Wei, Rongsheng Lu, Yifan Liu, Chenchen Xu, Haoran Cai, Yanhong Huang, Peng Li, Xiaoman Ye, Yong Li

**Affiliations:** ^1^ Department of Cardiology, The First Affiliated Hospital of Nanjing Medical University, Nanjing, Jiangsu, China; ^2^ Department of Clinical Medicine, The First Clinical Medical College of Nanjing Medical University, Nanjing, Jiangsu, China; ^3^ State Key Laboratory of Reproductive Medicine, School of Public Health, Nanjing Medical University, Nanjing, Jiangsu, China; ^4^ Jiangsu Key Laboratory for Design and Manufacture of Micro-Nano Biomedical Instruments, Southeast University, Nanjing, China; ^5^ Department of Intensive Care Medicine, The Fourth Affiliated Hospital of Nanjing Medical University, Nanjing, Jiangsu, China; ^6^ Department of Cardiology, The People's Hospital of Qijiang District, Qijiang, Chongqin, China

**Keywords:** chronic kidney disease, dialysis patients, MI occurrence, hs-cTnT, Albumin (Alb), triglyceride (TG)

## Abstract

**Background:**

As a sensitive diagnostic marker for myocardial infarction (MI) in people with normal renal function, elevated high sensitivity cardiac troponin T (hs-cTnT) was often found in chronic kidney disease (CKD) patients requiring dialysis. However, the accuracy of baseline hs-cTnT in the diagnosis of MI (including Type 1 MI (T1MI) and Type 2 MI (T2MI)) in dialysis patients is still controversial. The aim of this study was to retrospectively explore whether there were any clinical indices that could increase the predictive value of hs-cTnT on admission for MI occurrence in dialysis patients.

**Methods:**

Here, 136 patients with uremia who underwent regular dialysis with coronary angiography in the First Affiliated Hospital of Nanjing Medical University from August 2017 to October 2021 were enrolled. According to the coronary angiography results and the presence of clinical symptoms, the patients were divided into: (1). AMI group (*n* = 69; angiography positive) and Control group (*n* = 67; angiography negative); (2). T1MI group (*n* = 69; angiography positive), T2MI group (*n* = 7; angiography negative & symptomatic), and Control group (*n* = 60; angiography negative & asymptomatic).

**Results:**

Here, we found the mean hs-cTnT on admission in the Control group was much lower than that in the AMI group. Hs-cTnT alone had a mediocre predictive performance, with an AUROC of 0.7958 (95% CI: 0.7220, 0.8696). Moreover, the ROC curve of hs-cTnT combined with the Triglyceride (TG), Time of dialysis, and Albumin (Alb) showed a higher sensitivity area [0.9343 (95% CI: 0.8901, 0.9786)] than that of single hs-cTnT. Next, hs-cTnT combined with the TG, Time of dialysis, and Alb also presented a better performance in predicting T1MI [0.9150 (95% CI: 0.8678, 0.9621)] or T2MI (0.9167 [0.9167 (95% CI: 0.8427, 0.9906)] occurrences. Last, these combined variables could better distinguish patient between T1MI and T2MI group than hs-cTnT alone.

**Conclusions:**

On admission, a combination of hs-cTnT, TG, Time of dialysis, and Alb presented a higher sensitivity than hs-cTnT alone in predicting MI occurrence in dialysis patients, suggesting a better diagnostic approach for future clinical applications.

## Introduction

Chronic kidney disease (CKD), due to its incidence estimated to continuously grow, will bring a heavy global burden of disease (1, 2). Epidemiological study predicts that the number of dialysis patients in China will exceed 870,000 by 2025 (2). Cardiovascular disease (CVD), including acute myocardial infarction (AMI), is the most common cause of death for dialysis patients (3). Cardiac troponin T (hs-cTnT) can be used as a sensitive serological marker for the diagnosis of myocardial damage in people with normal renal function (4), but its levels vary across a considerable number of patients, who suffer end-stage renal disease (including dialysis patients), but show no clinical symptoms of MI (5, 6). At present, its prognostic significance in this patient population is still controversial.

In addition, serum hs-cTnT level increases nonlinearly with the deterioration of renal function, which makes it more difficult to predict the occurrence of MI in CKD patients (6). Also, a previous study has reported that hs-cTnT, just like tossing a coin, achieves a low accuracy in diagnosing MI in non-dialysis patients with renal insufficiency (7).

Here, we aimed to investigate the accuracy of baseline hs-cTnT in the diagnosis of MI in dialysis patients., and further explore whether any other clinical indices could increase the predictive value of hs-cTnT on admission.

## Material and methods

### Ethics statement and consent to participate

The clinic data of patients were collected according to the Declaration of Helsinki and the First Affiliated Hospital of Nanjing Medical University's ethics committee (No. 2023-SR-787). All the patients have been informed about this research, so that their written informed consent have be obtained in addition to other procedural safeguards.

### Study design and population

A retrospective study was conducted on 136 patients with uremia who underwent regular dialysis with coronary angiography in the First Affiliated Hospital of Nanjing Medical University from August 2017 to October 2021. Patients' age, medical history, comorbidities, and risk factors for coronary heart disease (e.g., hypertension, diabetes, hyperlipidemia) were detailed. Among the 136 patients [93 males and 43 females, age 28–86 years (mean 64.14 ± 12.07 years)], 116 had hypertension and 77 had diabetes. 1. According to the coronary angiography results, the patients were divided into angiography positive group (AMI group, *n* = 69) and angiography negative group (Control group, *n* = 67). 2. According to the coronary angiography results and the presence of clinical symptoms, the patients were divided into Type 1 MI (T1MI) group (*n* = 69; angiography positive), Type 2 MI (T1MI) group (*n* = 7; angiography negative & symptomatic), and Control group (*n* = 60; angiography negative & asymptomatic).

### Inclusion criteria

(1) Regular dialysis for uremia was performed in a period of over 6 months; (2) Blood hs-cTnT levels elevated; (3) The patient was accompanied with or without chest pain, chest tightness, dyspnea and other symptoms; (4) During dialysis, coronary angiography was performed to clarify coronary artery lesions.

All enrolled patients received coronary angiography for the following reasons: (1). Presented clinical signs of myocardial ischemia; (2). Abnormal cardiac markers; (3). Abnormal electrocardiogram results; (4). The required cardiovascular evaluation before surgery.

### Exclusion criteria

The patient had other diseases that may cause hs-cTnT elevation, such as acute pericarditis, acute myocarditis, cardiomyopathy, tachycardia, myocardial contusion, subarachnoid hemorrhage, acute pulmonary embolism, sepsis, or post-AMI, etc.

### Evaluation of coronary heart disease severity

Coronary angiography was performed by two experienced interventional cardiologists. Stenosis ≥50% was positive, and stenosis < 50% was negative. Since the well-known role of Gensini score in evaluating the severity of coronary atherosclerosis (8), Gensini score was calculated according to the location and degree of coronary stenosis in each patient. First, the basic score was determined according to the degree of coronary artery stenosis: diameter stenosis <25% was given a score of 1 point, ≥25%–<50% of 2 points, ≥50%–<75% of 4 points, ≥75%–<90% of 8 points, ≥90%–<99% of l6 points, and 99%–100% of 32 points. Then, the basic scores in different coronary branches were multiplied by the following coefficients: left main artery (LM) disease ×5; left anterior descending branch (LAD) disease, proximal segment ×2.5, middle segment ×1.5, distal segment ×1, diagonal branch disease D1 × 1, D2 × 0.5; left circumvolute branch (LCX) disease, proximal segment ×2.5, blunt margin branch ×1, distal segment ×1, posterior descending branch ×1, posterior lateral branch ×0.5; right coronary artery (RCA) lesions, proximal, middle, distal and posterior descending branches ×1. The scores of all diseased vessels were summed to indicate the severity of coronary heart disease in one patient.

### Physical and blood biochemical tests

Lung infection was evaluated based on the preoperative chest CT. After admission, the patient's resting blood pressure was measured by an electronic sphygmomanometer. Cubital venous blood was collected after 12 h of fasting before dialysis procedure. Measured were hs-cTnT, leukocyte, hemoglobin, serum creatinine (Scr), urea nitrogen (BUN), uric acid, serum Albumin (Alb), total cholesterol (TC), triglyceride (TG), high density lipoprotein cholesterol (HDL-C), low density lipoprotein cholesterol (LDL-C), electrolyte potassium, sodium, calcium, phosphorus and NT-proBNP levels. hs-cTnT was determined in serum using an Elecsys 2010 automated immunochemistry analyzer (Roche Diagnostics, Mannheim, Germany). LVEF level was evaluated by Simpson echocardiography. Serum levels of white blood cell, hemoglobin, Scr, BUN, uric acid, serum Alb, TC, TG, HDL-C, LDL-C, electrolyte potassium, sodium, calcium, phosphorus and NT-proBNP were measured. The level of LVEF was evaluated by Simpson method of cardiac echocardiography.

### Random forest algorithm to assess predictive values

We performed receiving operational curve (ROC) analysis and calculated the area under curve (AUC) to assess the predictive performance of the model with the “pROC” R package. An optimal cut-off value was determined based on the ROC analysis, and the sensitivity and specificity were calculated according to the cut-off value.

### Statistical analysis

Continuous variables were expressed by means ± standard deviations, and categorical variables by frequencies and percentages. The independent-samples *t* test was used to compare mean values in case and control groups. The chi-squared and Fisher exact test was used to describe qualitative data. hs-cTnT levels were log transformed, and partial correlation analysis was used to analyze the correlation coefficient between hs-cTnT level and influencing factors. The statistical significance level was set at *P* < 0.05. SPSS 20 statistical software was used to process the data.

## Results

### Patients' characteristics between AMI and control group

On admission, the epidemiological data, medical history, underlying comorbidities, and clinical symptoms of all the 136 dialysis patients were obtained with standardized forms. According to the results of coronary angiography, the dialysis patients were divided into the AMI group and the Control group. The AMI group (*n* = 69, 52 males, age 65.06 ± 10.82 years) was matched with the Control group (*n* = 67, 41 males, age 63.19 ± 13.25 years) in sex (*F* = 0.097, *P* = 0.097) and age (*F* = 0.81, *P* = 0.371). There were 49 patients (71%) with diabetes mellitus in the AMI group, which was significantly higher than that in the Control group (*P* = 0.001). The white blood cell count and uric acid level in the AMI group were significantly higher than those in the Control group (*P* = 0.016; *P* = 0.036), while the TG level, LVEF and dialysis time were significantly lower than those in the Control group (all *P* < 0.05; Table 1). Besides, the means of hs-cTnT were higher than the conventional reference in both groups. Nevertheless, the mean hs-cTnT in the Control group (100.35 ± 81.9) was much lower than that in the AMI group (1400.78 ± 2536.16) (*P* = 0).

**Table 1:** Characteristics of the study subjects.

Characteristic	Control (*n* = 67)	Case (*n* = 69)	*c*² or F	*P*
Age (years)	63.19 ± 13.25	65.06 ± 10.82	0.81	0.371
Sex (male/female)	41/26	52/17	0.097	0.097
SBP (mmHg)	141.6 ± 23.37	144.1 ± 22.12	0.398	0.529
DBP (mmHg)	78.36 ± 12.61	78.93 ± 12.61	0.061	0.805
cTnT	100.35 ± 81.9	1400.78 ± 2536.16	17.59	0*
White blood cell (×10^9^/L)	7.31 ± 3.13	8.86 ± 4.25	5.872	0.016*
Hemoglobin (g/L)	102.25 ± 21.26	98.10 ± 21.99	1.252	0.265
Scr (umol/L)	697.26 ± 226.98	625.6 ± 243.7	3.14	0.078
BUN (mmol/L)	20.5 ± 7.00	21.13 ± 7.30	0.275	0.601
Uric acid (umol/L)	330.84 ± 91.62	371.9 ± 130.46	4.479	0.036*
TG (mmol/L)	2.12 ± 1.88	1.50 ± 0.73	6.168	0.014*
TC (mmol/L)	3.94 ± 1.39	3.90 ± 1.39	0.051	0.822
HDL-C (mmol/L)	0.97 ± 0.27	0.93 ± 0.26	0.536	0.466
LDL-C (mmol/L)	2.38 ± 0.9	2.38 ± 0.98	0.009	0.924
Potassium (mmol/L)	4.34 ± 0.64	4.43 ± 0.63	0.598	0.441
Sodium (mmol/L)	138.64 ± 3.02	138.5 ± 4.06	0.043	0.836
Calcium (mmol/L)	2.24 ± 0.26	2.26 ± 0.24	0.075	0.785
Phosphorus (mmol/L)	1.74 ± 0.55	1.63 ± 0.42	1.858	0.177
NT-proBNP (pg/ml)	15973.41 ± 12316.96	19351 ± 12505	2.516	0.115
LVEF (%)	0.60 ± 0.07	0.53 ± 0.11	8.25	0.005*
Complication				
Hypertension (%)	54 (80.6%)	62 (89.9%)	0.151	0.151
Diabetes (%)	28 (41.8%)	49 (71%)	0.001	0.001*
Pulmonary infection (%)	26 (38.8%)	28 (40.6%)	0.862	0.862
Diabetic nephropathy (%)	17 (25.4%)	18 (26.1%)	1	1
Time of dialysis (years)	5.46 ± 5.12	3.45 ± 4.96	5.4	0.022*

* *P*-value < 0.05.

The level of hs-cTnT was converted to log hs-cTnT, and the correlation between log hs-cTnT and Gensini score or physical and chemical indexes was analyzed by partial correlation analysis. The results showed that the level of log hs-cTnT was positively correlated with Gensini score, NT-proBNP and white blood cell count (*r* = 0.364, *r* = 0.268, *r* = 0.326, *P* < 0.05), and negatively correlated with TG, serum Alb and LVEF (%) (*r* = −0.171, *r* = −0.171, *P* < 0.05). *r* = −0.313, *r* = −0.18, both *P* < 0.05), but the correlation was weak (Table 2).

**Table 2:** Correlation analysis between cTNT level and influencing factors.

Demographics	log cTnT	
	Correlation coefficient	*P* value
Age (years)	0.122	0.163
Gensini score	0.364	0*
SBP (mmHg)	−0.085	0.33
DBP (mmHg)	−0.066	0.454
White blood cell (×10^9^/L)	0.326	0*
Hemoglobin (g/L)	−0.089	0.31
Scr (umol/L)	−0.144	0.099
BUN (mmol/L)	0.154	0.076
Uric acid (umol/L)	0.081	0.357
TG (mmol/L)	−0.171	0.049*
TC (mmol/L)	0.049	0.576
HDL-C (mmol/L)	−0.041	0.639
LDL-C (mmol/L)	0.058	0.507
serum albumin (g/L)	−0.313	0*
Potassium (mmol/L)	0.15	0.085
Sodium (mmol/L)	0.02	0.817
Calcium (mmol/L)	0.024	0.78
Phosphorus (mmol/L)	0.079	0.366
NT-proBNP (pg/ml)	0.268	0.002*
LVEF (%)	−0.18	0.039*
Time of dialysis (years)	−0.073	0.408

* *P*-value < 0.05.

### ROC curve of hs-cTnT for AMI diagnosis on admission

**Figure 1:**
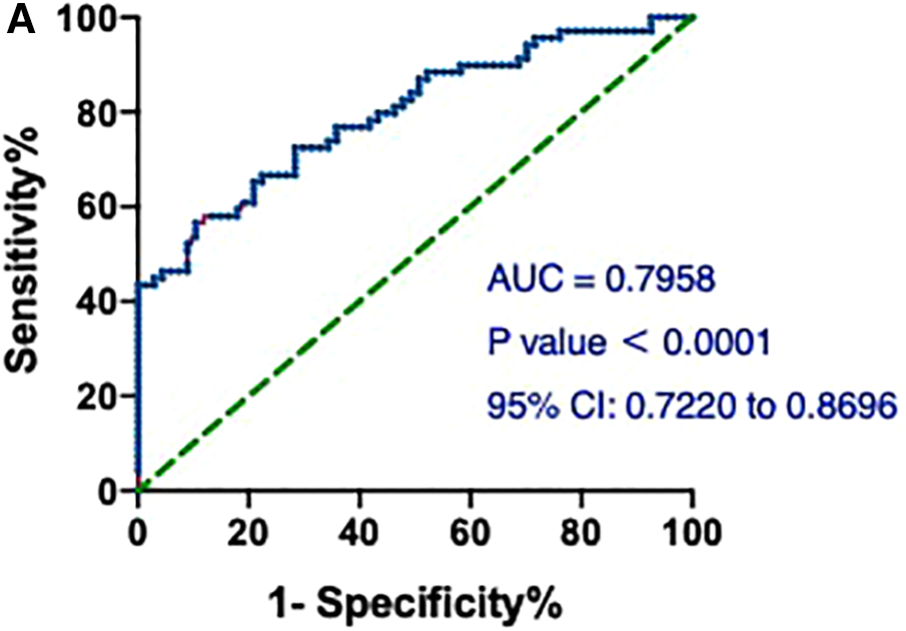
ROCs and AUC of hs-cTnT alone on admission in patients with AMI. A, hs-cTnT alone had a mediocre predictive performance, with an AUROC of 0.7958 (95% CI: 0.7220, 0.8696). The Random Forest algorithm was used to assess predictive values with the “pROC” R package.

First, the value of hs-cTnT on admission in predicting the occurrence of AMI in the patients included in our study was assessed. As shown in Figure 1, hs-cTnT alone had a mediocre predictive performance, with an AUROC of 0.7958 (95%CI: 0.7220, 0.8696).

**Figure 2:**
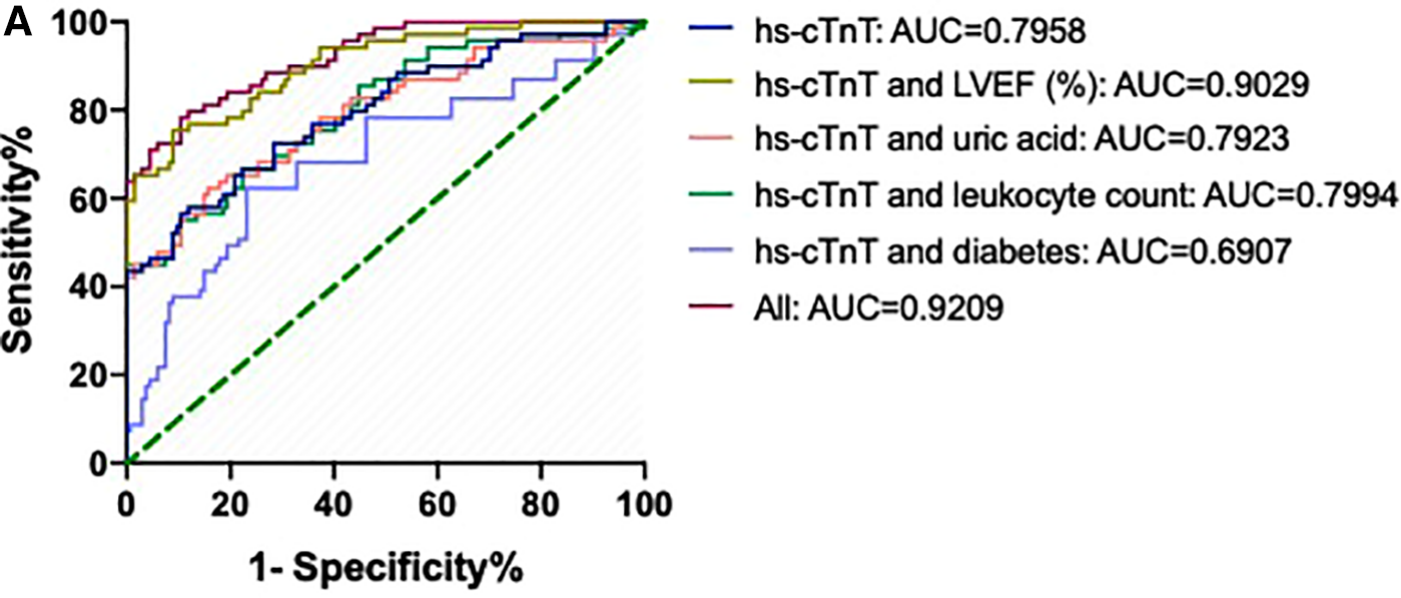
ROCs and AUCs of hs-cTnT combined with diabetes, leukocyte count, uric acid, and LVEF on admission in patients with AMI. Blue line: hs-cTnT alone; Light purple line: The combination of hs-cTnT and diabetes; Green line: The combination of hs-cTnT and leukocyte count; Pink line: The combination of hs-cTnT and uric acid; Golden line: The combination of hs-cTnT and LVEF (%); Diamond red line: The combination of hs-cTnT and diabetes, leukocyte count, uric acid, and LVEF. The Random Forest algorithm was used to assess predictive values with the “pROC” R package.

The areas under the ROC (AUCs) of hs-cTnT combined with diabetes, leukocyte count, uric acid, and LVEF (%) were 0.6907 (95% CI: 0.6009, 0.7804), 0.7994 (95% CI: 0.7263, 0.8725), 0.7923 (95% CI: 0.7173, 0.8674), and 0.9029 (95% CI: 0.8541, 0.9516), respectively (Figure 2). Interestingly, the AUC of a combination of hs-cTnT, diabetes, leukocyte count, uric acid, and LVEF (%) was 0.9209 (95% CI: 0.8789, 0.9630).

**Figure 3:**
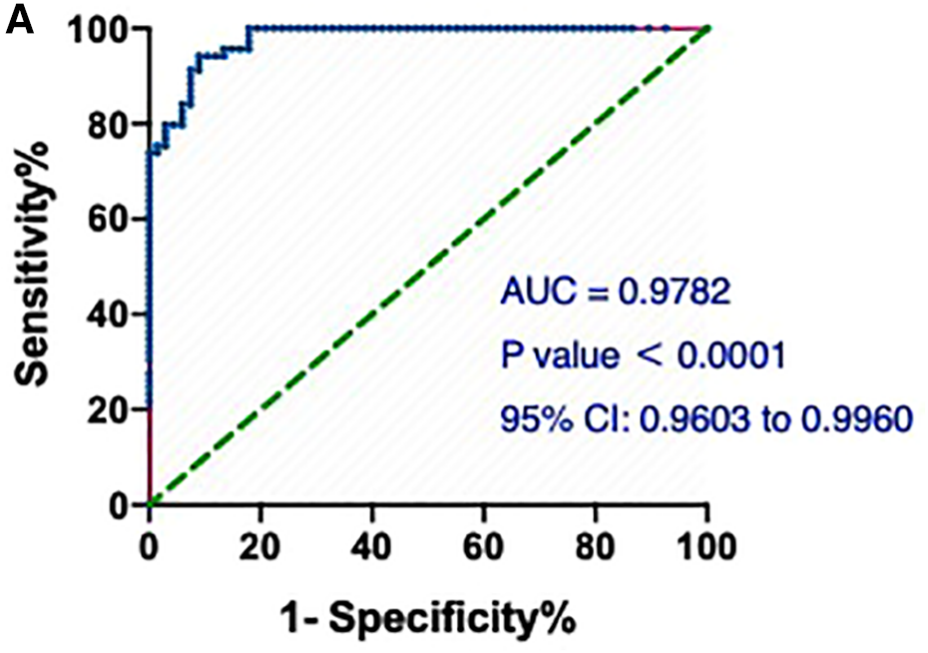
The ROC and AUC of hs-cTnT combined with other clinical variables on admission in patients with AMI. A, the model showed a better predictive performance when including the combination of hs-cTnT and other clinical variables shown in Table 1 (AUROC: 0.9782, 95% CI: 0.9603, 0.9960). The Random Forest algorithm was used to assess predictive values with the “pROC” R package.

**Figure 4:**
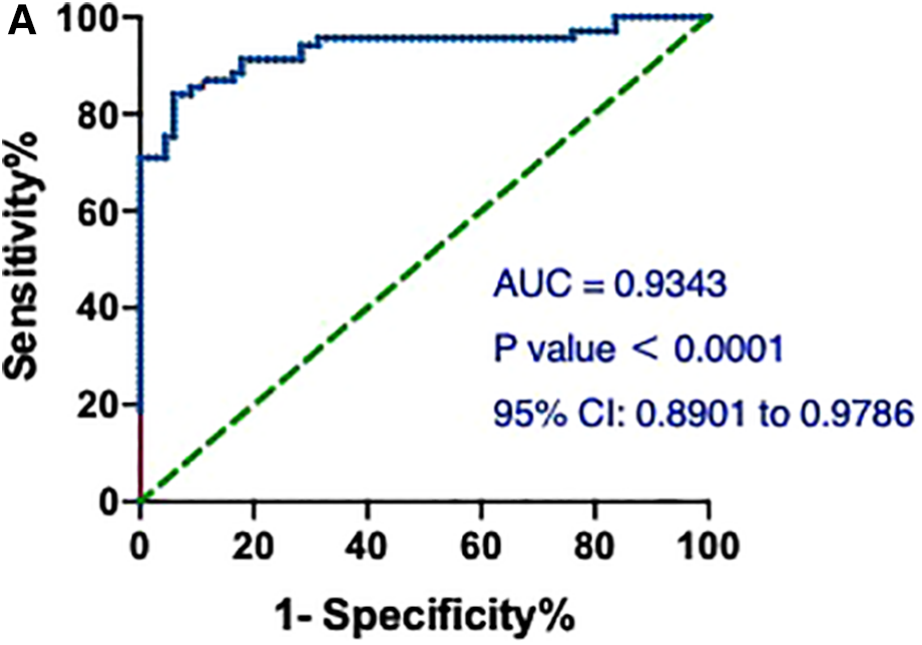
The ROC and AUC of hs-cTnT combined with the TG, time of dialysis (years) and Alb on admission in patients with AMI. A, the ROC curve of hs-cTnT combined with the TG, Time of dialysis, and Alb showed a higher sensitivity area [0.9343 (95% CI: 0.8901, 0.9786)] than that of single hs-cTnT. The Random Forest algorithm was used to assess predictive values with the “pROC” R package.

Notably, the model showed a better predictive performance when including the combination of hs-cTnT and other clinical variables shown in Table 1 (AUROC: 0.9782, 95% CI: 0.9603, 0.9960) (Figure 3). We created a Random Forest model in R software to assess the effects of these variables on the predictive ability of hs-cTnT on admission. The results showed that TG, Time of dialysis, and Alb were the top three variables with the highest Mean Decrease Gini (Table 3). Next, on admission, the ROC curve of hs-cTnT combined with the TG, Time of dialysis, and Alb showed a higher sensitivity area [0.9343 (95% CI: 0.8901, 0.9786)] than that of single hs-cTnT (Figure 4), indicating the diagnostic value of these combined variables.

**Table 3:** Effects of clinical variables on the predictive ability of cTnT.

Demographics	Mean Decrease Gini
Serum albumin (g/L)	1.852768063
Time of dialysis (years)	1.013308308
Triacylglycerol (mmol/L)	1.001560376
LVEF (%)	0.911744318
Phosphorus (mmol/L)	0.814853869
Diabetes	0.751358035
White blood cell (×10^9^/L)	0.667209224
LDL-C (mmol/L)	0.623972802
NT-proBNP	0.562943542
Hemoglobin (g/L)	0.542078011
Calcium (mmol/L)	0.533148112
Total cholesterol (mmol/L)	0.528054874
Age (years)	0.526987098
Uric acid (μmol/L)	0.519981876
Urea nitrogen (mmol/L)	0.516375421
Serum creatinine (μmol/L)	0.486196382
HDL-C (mmol/L)	0.459910766
Sodium (mmol/L)	0.450597923
Systolic pressure (mmHg)	0.408808899
Potassium (mmol/L)	0.399864695
Diastolic pressure (mmHg)	0.337324201
Hypertension (%)	0.205939855
Sex (male/female)	0.008855296

### Patients' characteristics between T1MI, T2MI and control group

According to the newly released “Fourth Universal Definition of Myocardial Infarction”, MI was classified into five types, the largest of which are T1MI and T2MI (9). Here, symptomatic patients with positive or negative angiographic results were enrolled in the T1MI o'r T2MI groups, respectively. The Control group (*n* = 60, 34 males, age 63.85 ± 13.49 years) was matched with the T1MI group (*n* = 69, 52 males, age 65.06 ± 10.82 years) and the T2MI group (*n* = 7, 7 males, age 57.57 ± 9.91 years) in age. However, there were significantly more males in T1MI and T2MI groups than in Control group (P_Control vs. T1MI _= 0.0388; P_Control vs. T2MI _= 0.0375). There were 49 and 6 patients with diabetes mellitus in the T1MI and T2MI group, respectively, which was significantly higher than that in the Control group (P_Control vs. T1MI _= 0.0001; P_Control vs. T2MI _= 0.0183). The White blood cell count and Uric acid level in the T1MI group were significantly higher than those in the Control group and T2MI group, while the TG level, Alb level, LVEF value and dialysis time were significantly lower than those in the Control group and T2MI (all *P* < 0.05; Table 4). Besides, the means of hs-cTnT were higher than the conventional reference in both groups. Nevertheless, the mean hs-cTnT in the T2MI group (1400.78 ± 2536.16) was much higher than that in the T2MI group (206.5 ± 77.56) and the Control group (87.97 ± 73.45) (*P* = 0.0001), while no significant difference of hs-cTnT was found between the T2MI and the Control group (*P* = 0.9826).

**Table 4:** Characteristics of the study subjects.

Characteristic	Control (*n* = 60)	T1MI (*n* = 69)	T2MI (*n* = 7)	P (Control vs. T1MI)	P (Control vs. T2MI)
Age (years)	63.85 ± 13.49	65.06 ± 10.82	57.57 ± 9.91	0.811	0.3443
Sex (male/female)	34/26	52/17	7/0	0.0388*	0.0375*
SBP (mmHg)	142.3 ± 23.82	144.1 ± 22.12	135.9 ± 19.57	0.8788	0.7259
DBP (mmHg)	78.45 ± 14.40	78.93 ± 12.61	77.57 ± 13.05	0.9739	0.9827
cTnT	87.97 ± 73.45	1400.78 ± 2536.16	206.5.78 ± 77.56	0.0001*	0.9826
White blood cell (×10^9^/L)	7.20 ± 3.15	8.86 ± 4.25	8.21 ± 2.97	0.0261*	0.7448
Hemoglobin (g/L)	102.4 ± 21.16	98.10 ± 21.99	101.4 ± 23.80	0.4594	0.9926
Scr (umol/L)	701.9 ± 221.9	625.6 ± 243.7	657.0 ± 283.2	0.132	0.8626
BUN (mmol/L)	20.38 ± 7.09	21.13 ± 7.30	21.48 ± 6.74	0.7931	0.9087
Uric acid (umol/L)	325.6 ± 92.94	371.9 ± 130.46	375.9 ± 68.89	0.0425*	0.4553
TG (mmol/L)	2.20 ± 1.93	1.50 ± 0.73	1.39 ± 0.97	0.0109*	0.2752
TC (mmol/L)	4.09 ± 1.36	3.90 ± 1.39	2.89 ± 0.97	0.6751	0.0559
HDL-C (mmol/L)	0.96 ± 0.29	0.93 ± 0.26	0.83 ± 0.12	0.7271	0.4072
LDL-C (mmol/L)	2.47 ± 0.88	2.38 ± 0.98	1.64 ± 0.63	0.8053	0.0518
serum albumin (g/L)	37.61 ± 4.60	33.63 ± 4.73	33.46 ± 4.44	0.0001*	0.0528
Potassium (mmol/L)	4.30 ± 0.63	4.43 ± 0.63	4.61 ± 0.49	0.4574	0.3855
Sodium (mmol/L)	138.5 ± 3.00	138.5 ± 4.06	139.3 ± 3.15	0.9996	0.8207
Calcium (mmol/L)	2.29 ± 0.21	2.26 ± 0.24	2.21 ± 0.24	0.6373	0.5686
Phosphorus (mmol/L)	1.74 ± 0.53	1.63 ± 0.42	1.73 ± 0.65	0.3201	0.9982
NT-proBNP (pg/ml)	16515 ± 12361	19351 ± 12505	11330 ± 11755	0.35	0.4988
LVEF (%)	0.61 ± 0.07	0.53 ± 0.11	0.56 ± 0.10	0.0001*	0.4697
Complication					
Hypertension (%)	48 (80.0%)	62 (89.9%)	6 (85.7%)	0.1389	>0.9999
Diabetes (%)	22 (36.7%)	49 (71%)	6 (85.7%)	0.0001*	0.0183*
Pulmonary infection	24 (40.0%)	28 (40.6%)	2 (28.6%)	>0.9999	0.6972
Diabetic nephropathy (%)	14 (23.3%)	18 (26.1%)	3 (42.9%)	0.8386	0.358
Time of dialysis (years)	5.71 ± 4.88	3.45 ± 4.96	3.29 ± 6.97	0.0236*	0.4005

* *P*-value < 0.05.

### ROC curve of hs-cTnT for T1MI and T2MI diagnosis on admission

**Figure 5:**
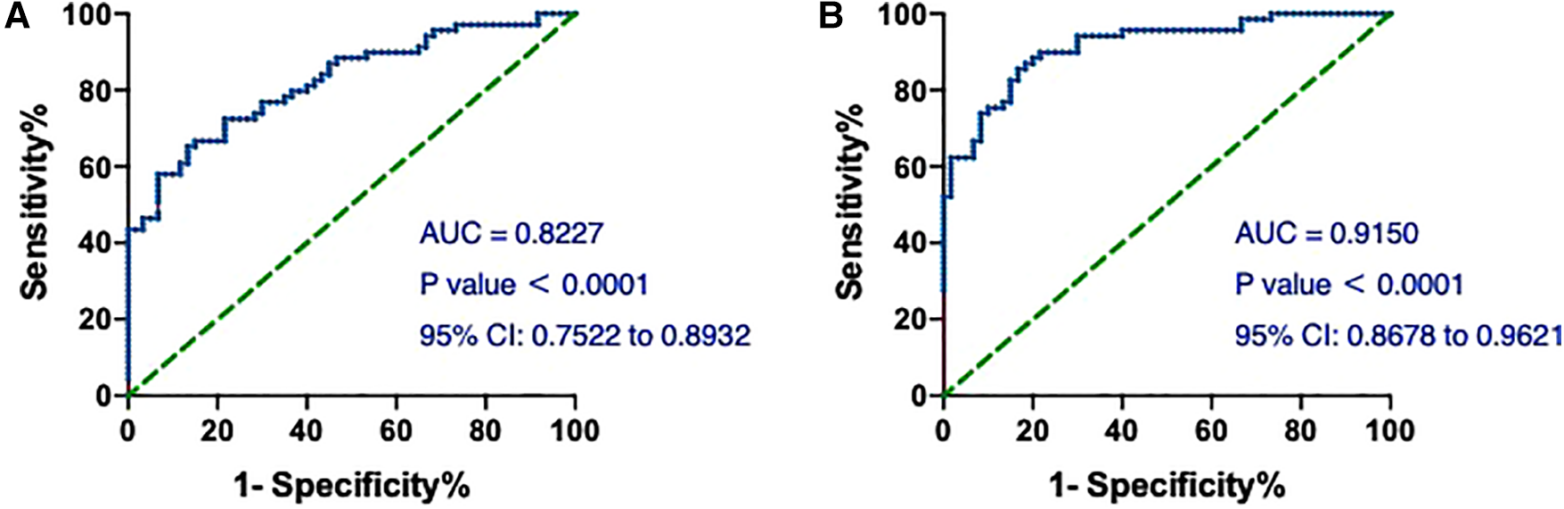
The ROC and AUC of hs-cTnT on admission in T1MI patients. (**A**) The ROC curve of hs-cTnT alone; (**B**) the ROC curve of hs-cTnT combined with the TG, Time of dialysis, and Alb. The Random Forest algorithm was used to assess predictive values with the “pROC” R package.

Then, we assessed the value of hs-cTnT on admission in predicting the occurrence of T1MI in the patients. As shown in Figure 5A, the AUCs of hs-cTnT alone were 0.8227 (95% CI: 0.7522, 0.8932). After combined with the top 3 variables (TG, Time of dialysis, and Alb) which generated from the Mean Decrease Gini data (Table 3), hs-cTnT showed a better predictive performance, with an AUROC of 0.9150 (95% CI: 0.8678, 0.9621) (Figure 5B).

**Figure 6:**
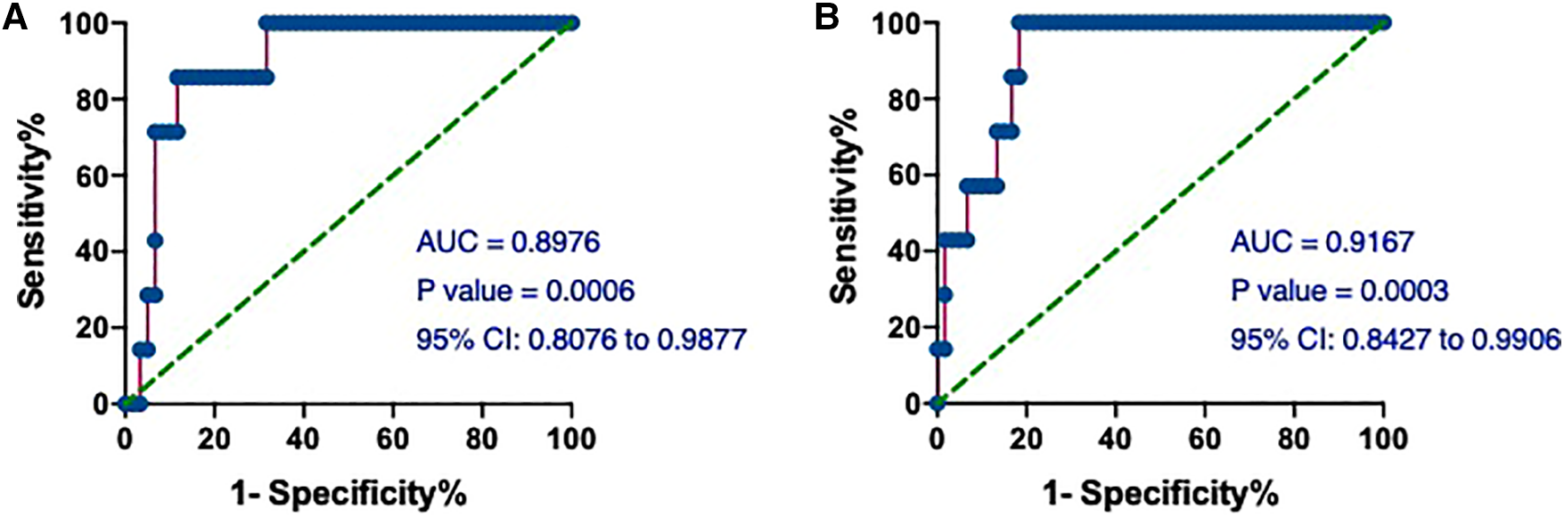
The ROC and AUC of hs-cTnT on admission in T2MI patients. (**A**) The ROC curve of hs-cTnT alone; (**B**) the ROC curve of hs-cTnT combined with the TG, Time of dialysis, and Alb. The Random Forest algorithm was used to assess predictive values with the “pROC” R package.

We next assessed the value of hs-cTnT on admission in predicting the occurrence of T2MI. The AUCs of hs-cTnT alone were 0.8976 (95% CI: 0.8076, 0.9877) (Figure 6A). Meanwhile, the ROC curve of hs-cTnT combined with the TG, Time of dialysis, and Alb showed a higher sensitivity area [0.9167 (95% CI: 0.8427, 0.9906)] than that of single hs-cTnT (Figure 6B).

**Figure 7:**
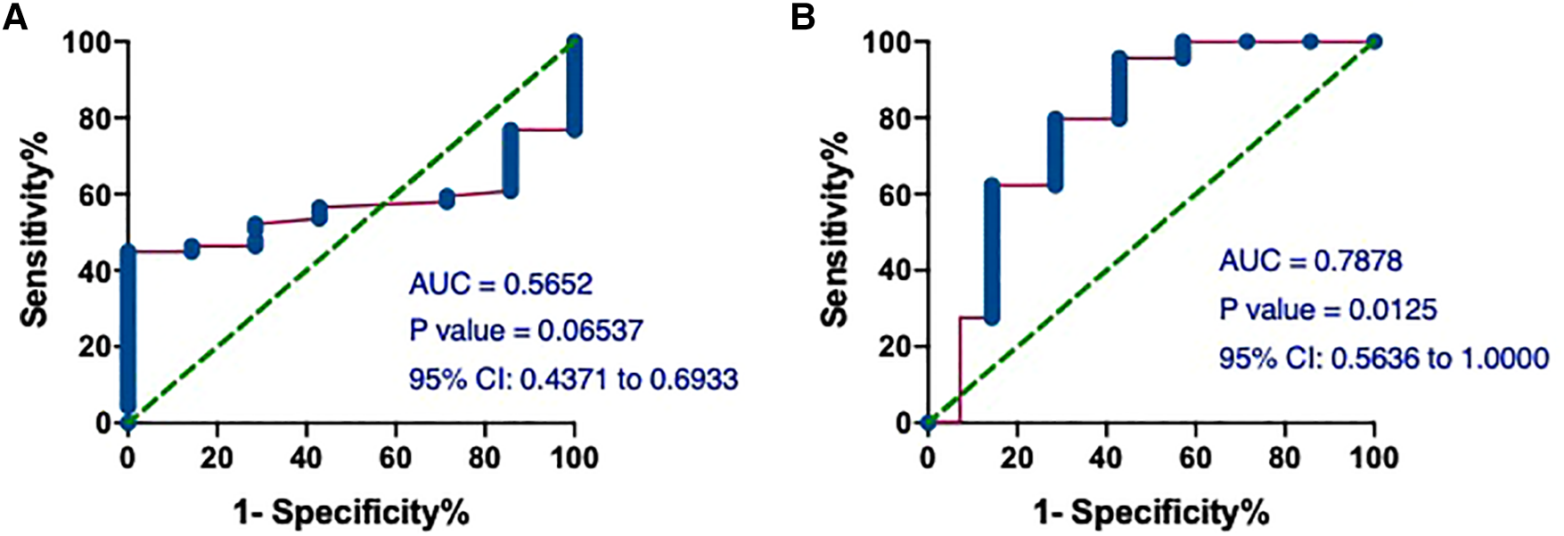
The ROC and AUC of hs-cTnT on admission between patients in T1MI and T2MI groups. (**A**) The AUCs of hs-cTnT alone were 0.5652 (*P* = 0.06537). (**B**) The ROC curve of hs-cTnT combined with the TG, Time of dialysis, and Alb showed a higher sensitivity area [0.7878 (95% CI: 0.5636, 1.000)]. The Random Forest algorithm was used to assess predictive values with the “pROC” R package.

Given the difference in mean hs-cTnT values between the T1MI and T2MI groups (Table 4), we performed ROC analysis and calculated the AUCs to assess the predictive performance of the model in distinguishing between patients in these 2 groups. The AUCs of hs-cTnT alone were 0.5652 (*P* = 0.06537) (Figure 7A). Notably, on admission, the ROC curve of hs-cTnT combined with the TG, Time of dialysis, and Alb showed a higher sensitivity area [0.7878 (95% CI: 0.5636, 1.000)] (Figure 7B).

## Discussion

The large population of CKD in China, coupled with several “blocking points” in prevention and control, such as inadequate detection ability at the grassroots level, interaction with cardiovascular and metabolic diseases, and increasing number of end-stage patients requiring dialysis, will result in a greater public health burden in the future continuously, which requires urgent attention (1).

In this study, we for the first time found that hs-cTnT on admission, especially combined with some clinical variables, was sensitive to predict AMI in dialysis patients. In addition, it is often found that the CKD patients presenting with chest pain, though accompanied without AMI, have a permanently elevated high-sensitivity cardiac troponin (hs-cTn) (10, 11). Consistently, we found that the dialysis patients enrolled in our study, either with or without AMI, had hs-cTnT levels higher than the conventional reference.

As a protein mainly existing in the complex of hs-cTnT-cTnI-cTnC of cardiomyocyte filaments, hs-cTnT is commonly used as a biomarker for the diagnosis of acute coronary events (12). Specifically, when myocardial cells are damaged due to ischemia and hypoxia, hs-cTnT is unbound and released rapidly from the cells into the bloodstream, which may explain why hs-cTnT appears earlier in circulating blood and persists for a long period in diseases characterized by damage to cardiomyocytes, such as AMI (13, 14). It is reported that the sensitivity of hs-cTnT reaches more than 90% within 6 h after AMI onset and maintained for more than 5 days.

Although hs-cTnT is often used as a marker of AMI occurrence, its elevation is not specific. The fact that hs-cTnT is often higher than the conventional reference in other non-coronary diseases (including renal insufficiency) poses a great clinical challenge for physicians (5, 6). In our study, a large proportion of dialysis patients with elevated hs-cTnT levels did not have AMI. Several explanations have been proposed for the elevated hs-cTnT levels in patients with impaired renal function: (1) redistribution of hs-cTnT expression in striated muscle in patients with CKD; (2) antigen cross reaction; (3) myocardial microdamage by chronic renal insufficiency.

First, PCR can be used to detect the abnormal expression of hs-cTnT in patients with chronic renal insufficiency, which denies the first hypothesis (15). Then, the second generation hs-cTnT detection method can avoid antigen cross reaction (16, 17). Last, most scholars believe that the elevated serum hs-cTnT level in CKD patients is a sign of sustained damage or even apoptosis of cardiomyocytes caused by uremic toxin or complications (18). Advanced renal insufficiency, along with diabetes mellitus, is even regarded as an independent risk factor for ischemic heart diseases. Heart failure and ventricular remodeling, which are commonly complicated by CKD, may result in insufficient subendocardial perfusion and abnormal troponin release. Meanwhile, uremic toxin-induced uremic pericarditis, uremic myocarditis and uremic cardiomyopathy may be secondary to elevated serum troponin levels. In addition, population-based cohorts (19, 20) and pathological studies (21, 22) found that the estimated glomerular filtration rate (eGFR) was negatively correlated with the incidence of coronary atherosclerosis, and microvascular and macrovascular calcification. Asymptomatic myocardial ischemia or myocardial necrosis caused by these diseases may also cause the release of hs-cTnT from the myocardium into the bloodstream. Recent evidence suggests that the inflammatory response in patients with end-stage renal diseases may accelerate myocardial damage (23).

In addition to abnormal necrosis-unrelated release, impaired renal clearance provides a possibility to explain the elevated troponin in CKD patients. Free hs-cTnT, hs-cTnT-cTnI-cTnC complex and some hs-cTnT fragments are released into the bloodstream after myocardial damage. The relative molecular weight of hs-cTnT is 37 kDa, and that of hs-cTnT-cTnI-cTnC complex is 77 kDa. Healthy human kidneys can clear away cleaved hs-cTnT fragments (24). However, when renal function is impaired, decreased eGFR leads to the accumulation of hs-cTnT fragments in the body, which is manifested as an increase in serum hs-cTnT level (24). The rapid decline of serum hs-cTnT level after kidney transplantation can support this explanation (25).

Wayand et al. showed that the increase of serum hs-cTnT after hemodialysis was related to the concentration of blood after dialysis, but not with dialysis membrane and dialysis mode (26). Other scholars have suggested that hypotension and myocardial stunning during dialysis may also cause myocardial damage (27). Non-traditional risk factors, including uremic toxins, can also elevate infarct-unrelated troponin in uremic patients who need dialysis (28).

Therefore, hs-cTnT elevation is more accurate to predict acute or chronic myocardial injury, but does not necessarily indicate the occurrence of AMI. Nevertheless, an elevated hs-cTnT is strongly associated with poorer clinical outcomes and a higher mortality in CKD patients, no matter whether they are receiving dialysis or not (29). The US Food and Drug Administration has also endorsed the use of hs-hs-cTnT measurement for risk stratification in dialysis patients (30). Higher level of hs-cTnT was also linked to greater risk of long-term major adverse cardiovascular events (MACEs) (31). Indeed, a great difference in the AMI and the Control group for the values of hs-cTnT was observed. Besides, the predictive performance of hs-cTnT alone on admission for AMI was 0.7958 (95% CI: 0.7220, 0.8696) in our study, which is not too low. In addition, even in asymptomatic dialysis patients with or without known coronary diseases, temporal changes in hs-cTnT has been shown to be beneficial in predicting all-cause mortality, cardiovascular death, and sudden cardiac death independently (32, 33). Taking into consideration the fact that the huge differences of individual baseline hs-cTnT levels among dialysis patients (34), it is becoming increasingly important to better understand and quantify the expected temporal change of hs-cTNT over time, especially for patients with increased risks of CVDs. Though some authors preferred to increase the troponin threshold that signal MI (34), it would be better to check hs-cTnT level regularly in stable asymptomatic dialysis patients every 1–3 months or in cardiac symptomatic dialysis patients every 1–3 h to more rapidly rule-in and rule-out cases of MI. However, due to patients' compliance and economic conditions, we were not able to perform long-term follow-up of cTnT before and after PCI surgery or even after discharge for every enrolled patient, which remains to be further explored in our future investigation. Nevertheless, we explored other clinical indicators that could increase the sensitivity of hs-cTnT to predict the occurrence of AMI. Here, we ound that hs-cTnT combined with TG, Time of Dialysis (years), and Alb on admission showed a higher sensitivity than single hs-cTnT.

The low serum Alb level with high diagnostic sensitivity and specificity for ACS has attracted considerable attention (35). As a powerful predictor of all-cause mortality in patients with ACS (36), serum Alb level is initially proposed as an independent predictor of MACEs (37). Recent studies also reported that the CRP-Alb ratio or ischemia-modified Alb (IMA) is associated with high thrombus burden in patients with MI (38, 39). In addition, the serum Alb level was correlated significantly with cTnT levels in patients with acute ischemic stroke (AIS) (40). The combination of hs-cTnT, serum Alb, and other clinical variables allowed a risk distinction for morbidity in heart failure with preserved ejection fraction (HFpEF) patients (41).

Previous studies also revealed that serum TG was independently associated with the occurrence of ACS and the risk of coronary heart disease (CHD) recurrence, which may be important for risk stratification and management of patients before and after ACS occurrence (42, 43). Besides, in patients with renal dysfunction, TG correlated with cTnT may be a renal risk parameter (44). In addition, the TG-glucose index (TyG index) was regarded as a non-linear and reliable predictor of MACE in patients with ACS (45).

Recently, novel hs-cTnT assays, which permit the detection of low levels of cTnT, indeed improved diagnostic sensitivity of patients with suspected AMI in the hospital setting. However, when applied to individuals with factors associated with higher levels of cTnT, including CKD, the test results may be less specific. Moreover, the false-positive diagnosis of AMI would lead to more unnecessary intensive treatment like percutaneous intervention (PCI) surgery, which brings heavy economic burden to the family and society, and causes great waste of medical resources. Thus, novel approach integrating more clinic indexes with hs-cTnT to improve the diagnostic accuracy of MI (including T1MI and T2MI) is needed. In the present study, an encouraging result we found is that cTnT combined with TG, Time of Dialysis (years), and Alb on admission had a higher predictive value, which may help in the early prevention and cure of the sudden cardiac death or other adverse cardiovascular outcomes for patients with MI, and further provide theoretical basis for our subsequent clinical cohort study.

Last, as a subset of ACS, MI is classified into five types according to the established “Fourth Universal Definition of Myocardial Infarction” which released by the Joint European Society of Cardiology (ESC)/American College of Cardiology (ACC)/American Heart Association (AHA)/World Heart Federation (WHF) Task Force (9), which subsequently increases the awareness and knowledge about a surge of suspicious MI cases. The pathogenetic mechanisms underlying five types of MI differs widely. T1MI, the most common type of MI, is defined as ischemic necrosis of cardiomyocytes secondary to coronary thrombosis. T2MI occurs due to the imbalanced oxygen supply and/or demand induced by pathological conditions other than acute plaque change in the coronary vasculature (46). The last 3 types of MI are reportedly less than 5% of the total MI cases, including cardiac death (9). The distinct demographics between T1MI and T2MI are obviously different (47). Notably, T2MI occurs frequently among the elderly with multiple comorbidities and high-risk cardiovascular profiles, and therefore has a poorer prognosis than T1MI (47). Till now, since no significant differences of clinical signs and symptoms between T1MI and T2MI, effective and timely diagnosis of T2MI remains challenging, which entails accurate prevalently angiography (48). However, compared to the invasive and expensive angiography, additional evidence-based patient-tailored therapeutic means of T2MI were warranted. Like the previous study (49), our analysis found that the value of hs-cTnT in T1MI was significantly higher than that in T2MI. Notably, compared with cTnT alone, cTnT combined with TG, Time of Dialysis (years), and Alb could not only better predict the occurrence of T1MI and T2MI, but also better distinguish T1MI and T2MI in our study. However, further cohort studies should be well-designed to evaluate whether diagnostic algorithms based on clinical symptoms and hs-cTnT values could improve the differential diagnosis among coronary events from non-coronary sources of MI, as well as between T1MI and T2MI.

## Limitations

The number of enrolled patients' needs to be further increased. At the same time, data from more centers could have been included in this study, which would strongly support our results. In addition, the enrolled patients need to be followed up for a longer period of time to clarify the effect of hs-cTnT on the long-term outcome of hemodialysis patients.

## Conclusions

A higher serum hs-cTnT level may be more predictive of AMI occurrence. On admission, a combination of hs-cTnT, TG, Time of Dialysis (years), and Alb presents a higher sensitivity than single hs-cTnT. The diagnostic value of these combined variables should be further evaluated before clinical application.

## Data Availability

The original contributions presented in the study are included in the article/Supplementary Material, further inquiries can be directed to the corresponding authors.
